# Quality of life after laparoscopic surgery for very low rectal cancer: A sub‐analysis of the ultimate trial

**DOI:** 10.1111/codi.70255

**Published:** 2025-10-06

**Authors:** Hiroki Hamamoto, Keitaro Tanaka, Yuichiro Tsukada, Jun Watanabe, Yosuke Fukunaga, Yasumitsu Hirano, Kazuhiro Sakamoto, Masanori Yoshimitsu, Hisanaga Horie, Nobuhisa Matsuhashi, Yoshiaki Kuriu, Shuntaro Nagai, Madoka Hamada, Shinichi Yoshioka, Shinobu Ohnuma, Tamuro Hayama, Koki Otsuka, Yusuke Inoue, Kazuki Ueda, Yuji Toiyama, Satoshi Maruyama, Shigeki Yamaguchi, Motoko Suzuki, Toshihiro Misumi, Takeshi Naitoh, Masahiko Watanabe, Masaaki Ito, Ultimate Trial Group in Japan Society of Laparoscopic Colorectal Surgery, Manabu Shiozawa, Mitsuyoshi Tei, Masafumi Inomata, Takashi Nonaka, Heita Ozawa, Seiichiro Yamamoto, Yasuyuki Miyakura, Akio Shiomi, Mamoru Uemura, Hideaki Nishigori, Koya Hida, Masanori Hotchi, Shu Okamura, Shuji Saito, Hiroaki Nagano, Ryo Inada, Kohei Murata, Atsushi Tsuruta, Hirokazu Suwa, Yusuke Takahashi, Ryoji Makizumi, Yukihide Kanemitsu, Manabu Yamamoto, Shinichiro Mori

**Affiliations:** ^1^ Department of General and Gastroenterological Surgery Osaka Medical and Pharmaceutical University Osaka Japan; ^2^ Department of General, Breast and Digestive Surgery Otsu City Hospital Otsu Japan; ^3^ Department of Colorectal Surgery National Cancer Center Hospital East Chiba Japan; ^4^ Department of Surgery, Gastroenterological Center Yokohama City University Medical Center Yokohama Japan; ^5^ Department of Gastroenterological Surgery Cancer Institute Hospital, Japanese Foundation of Cancer Research Tokyo Japan; ^6^ Department of Gastroenterological Surgery Saitama Medical University International Medical Center Hidaka Japan; ^7^ Department of Coloproctological Surgery Juntendo University Faculty of Medicine Tokyo Japan; ^8^ Department of Surgery Hiroshima City North Medical Center Asa Citizens Hospital Hiroshima Japan; ^9^ Department of Surgery Jichi Medical University Tochigi Japan; ^10^ Department of Gastroenterological Surgery, Pediatric Surgery, Graduate School of Medicine Gifu University Gifu Japan; ^11^ Department of Surgery Kyoto Prefectural University of Medicine Kyoto Japan; ^12^ Department of Surgery and Oncology, Graduate School of Medical Sciences Kyushu University Fukuoka Japan; ^13^ Department of Gastrointestinal Surgery Kansai Medical University Hospital Hirakata Japan; ^14^ Department of Surgery Yao Municipal Hospital Osaka Japan; ^15^ Department of Surgery Tohoku University Hospital Sendai Japan; ^16^ Department of Surgery Teikyo University School of Medicine Tokyo Japan; ^17^ Department of Surgery Iwate Medical University School of Medicine Iwate Japan; ^18^ Department of Surgery Nagasaki University Graduate School of Biomedical Sciences Nagasaki Japan; ^19^ Division of Endoscopic & Colorectal Surgery, Department of Surgery Kindai University Faculty of Medicine Osaka Japan; ^20^ Department of Gastrointestinal and Pediatric Surgery Mie University Tsu Japan; ^21^ Department of Gastroenterological Surgery Niigata Cancer Center Hospital Niigata Japan; ^22^ Division of Gastrointestinal Surgery, Department of Surgery Tokyo Women's Medical University Tokyo Japan; ^23^ Department of Data Science National Cancer Center Hospital East Chiba Japan; ^24^ Department of Lower Gastrointestinal Surgery Kitasato University School of Medicine Sagamihara Japan; ^25^ Department of Surgery Kitasato University Kitasato Institute Hospital Tokyo Japan

**Keywords:** quality of life, SF‐36 questionnaire, very low rectal cancer

## Abstract

**Aim:**

Laparoscopic low anterior resection is widely used for treating rectal cancer; however, postoperative complications frequently result in a marked deterioration in the patients' quality of life (QOL). This study aimed to investigate the trajectory of QOL recovery and identify the risk factors affecting QOL in patients undergoing laparoscopic surgery for very low rectal cancer.

**Method:**

This prospective, multi‐institutional, single‐arm, phase‐II trial examined the outcomes of laparoscopic surgery in patients with very low rectal cancer (<5 cm from the anus). The Short Form 36 Health Survey questionnaire was used to assess QOL preoperatively and at 3 and 6 months and 1, 2 and 3 years postoperatively. Univariate and multivariate logistic regression analyses were performed to identify the risk factors for impaired QOL.

**Results:**

Overall, 259 patients were analysed, of whom 63% underwent intersphincteric resection. The physical component summary (PCS) score declined postoperatively but gradually recovered, whereas the mental component summary score showed sustained improvement. The role component summary (RCS) score significantly declined at 3 months but improved over time. Multivariate analysis identified stage III cancer as a risk factor for prolonged PCS decline (*p* = 0.0084) and Wexner score ≥9 at 1 year after surgery as a predictor of lower RCS score (*p* = 0.0116).

**Conclusion:**

QOL after laparoscopic surgery for low‐lying rectal cancer is generally acceptable. Patients with stage III cancer experience prolonged physical challenges, whereas those with bowel dysfunction struggle with role/social domains of QOL. Targeted interventions should be implemented to address these issues.

**What does this paper add to the literature?**

This study presents a 3‐year longitudinal QOL assessment after laparoscopic surgery for very low rectal cancer using the SF‐36. It identifies the key risk factors affecting physical and role/social recovery, emphasizing the need for tailored postoperative care, multidisciplinary support, and optimized chemotherapy strategies to improve patient outcomes.

## INTRODUCTION

Colorectal cancer remains one of the most prevalent malignancies worldwide, accounting for a significant proportion of colorectal cancer cases. Surgical intervention, specifically low anterior resection (LAR), is a common treatment aimed at achieving oncological control while preserving the anal sphincter function. Furthermore, the development of intersphincteric resection (ISR) by Schiessel et al. [1, 2, 3] in 1994 promoted sphincter preservation surgery. However, a major challenge following surgery for low‐lying rectal cancer is postoperative bowel dysfunction, which manifests as increased bowel movement frequency, urgency, incontinence, and a sense of incomplete evacuation. These symptoms can significantly affect daily life and overall well‐being [4, 5, 6, 7], highlighting the importance of comprehensive quality of life (QOL) assessments in this patient population. Moreover, bowel dysfunction tends to worsen in patients with tumours located closer to the anus. A systematic review and meta‐analysis identified tumour distance from the anal margin as a significant risk factor for severe dysfunction [8, 9].

The Short Form 36 Health Survey (SF‐36) questionnaire (Quality Metric, Lincoln, Rhode Island, USA) is a widely recognized tool that can effectively measure QOL. It evaluates various health domains, including physical functioning, bodily pain, general health, vitality, social functioning, role limitations due to physical health problems, role limitations due to emotional problems, and mental health [10]. The SF‐36 questionnaire is particularly valuable in capturing the multifaceted impact of chronic conditions and surgical outcomes on patients' lives. Despite its broad application in evaluating health conditions after rectal surgery [11, 12, 13, 14], there is a paucity of longitudinal studies specifically utilizing the SF‐36 questionnaire to assess QOL in patients after very low rectal cancer resection.

This study aimed to fill this gap by conducting a 3‐year longitudinal assessment of QOL in patients who underwent laparoscopic surgery for very low rectal cancer using the SF‐36 questionnaire. By systematically evaluating QOL at multiple time points, this study aimed to delineate the trajectory of QOL recovery by focusing on physical, mental, and social functions. Furthermore, this study investigated the key factors influencing QOL, providing deeper insights into the challenges faced by these patients.

## METHOD

This was a prospective, multi‐institutional, non‐randomized, single‐arm phase‐II trial investigating the outcomes of laparoscopic surgery in patients with very low rectal cancer located near (<5 cm) the anus. The inclusion and exclusion criteria for this study have been outlined in our initial report [15]. Briefly, the inclusion criteria were as follows: (1) clinical stage of T1–T2, N0, and M0 (Tumour, Node, Metastasis classification); (2) lower border of the tumour being located within 5 cm of the anal verge (AV) or 3 cm from the dentate line; and (3) having undergone laparoscopic intersphincteric or very low anterior resection. The study protocol was approved by the Ethics Committee of the Japanese Society for Cancer of the Colon and Rectum and was approved and overseen by the institutional review board of each participating hospital. Written informed consent was obtained from all the participants. This study was registered in the Japan Registry of Clinical Trials (UMIN000011750).

### QOL assessment

The Japanese version of the SF‐36 questionnaire was used for the general evaluation of QOL in this study. The SF‐36 comprises eight multi‐item scales: physical function, role limitations due to physical health problems, bodily pain, general health, vitality, social functioning, role limitations due to emotional health problems, and mental health. Compartment summary scores are calculated based on these subscales to provide global measures of physical function (physical component summary, PCS), mental function (mental component summary, MCS), and a role component summary (RCS). The scale scores range from 0 to 100, with higher scores indicating a better health status. These are norm‐based scores, with an average score of 50 for the population.

The decision to perform diverting ileostomy was based on the surgeon's technical evaluation of anastomosis quality. The SF‐36 questionnaire was administered to all the patients before surgery. It was administered postoperatively at 3 and 6 months and 1, 2, and 3 years after stoma closure for patients with a diverting stoma. In contrast, for patients without diverting stomas, the questionnaire was administered at 3 and 6 months and 1, 2, and 3 years after rectal resection.

### Subgroup analysis

To further explore the factors influencing QOL, we conducted subgroup analyses based on surgical method (ISR vs. LAR), distance of the tumour from the AV (<25 mm vs. ≥25 mm), pathological stage (III vs. 0–II), and the Wexner score at 1 year (≥9 vs. <8). The decision to categorize patients based on the Wexner score at 1 year was based on evidence suggesting that bowel function stabilizes around this time following rectal cancer surgery, making it a reliable indicator of long‐term dysfunction [16, 17].

### Statistical analysis

Statistical analysis was performed using SAS® 9.4 (SAS Institute, Cary, NC, USA). The mean values of the collected data were used to generate the line graphs. Multivariate analysis was performed using a mixed‐effects model, with the patient as the random effect and the surgical method, pathological stage, and the Wexner score at 1 year as fixed effects. Statistical significance was set at *p* < 0.05.

## RESULTS

### Patient characteristics and operative and pathological results

Eligible patients were enrolled from January 2014 to March 2017. Of the 299 patients who participated in this study, 259 patients returned the SF‐36 questionnaires and were included in the QOL analysis (Figure S1). The details of the 259 cases are presented in Table 1. They were recruited from 47 specialized centers in Japan. Of these, 63% were male and 37% were female. The median distance of the tumour from the AV was 40 mm. The clinical T stage was T1 in 59% and T2 in 41% of the patients. The surgical procedure was LAR in 37% and ISR in 63% of patients. A diverting stoma was created in 238 patients (92%). Among these, 230 patients (97%) underwent stoma closure, and 212 patients (92%) had their stoma closed within 1 year after the initial surgery.

**TABLE 1:** Patient characteristics, operative/pathological findings and postoperative outcomes.

	Patients (*n* = 259)
Patient characteristics	
Sex	
Male	162 (63%)
Female	97 (37%)
Age (years)	64 (30–87)
BMI (kg/m^2^)	22.8 (16.4–34.0)
Tumour from AV (mm)	40 (10–55)
Differentiation	
Well	160 (62%)
Moderate	89 (34%)
Others	10 (4%)
Clinical T stage	
T1	152 (59%)
T2	107 (41%)
Operative/pathological findings	
Laparoscopic procedures	
Low anterior resection	96 (37%)
Intersphincteric resection	163 (63%)
Operating time (min)	284 (113–656)
Blood loss (mL)	30 (0–740)
Diverting stoma	238 (92%)
Pathological T stage	
Tis	4 (1.5%)
T1	140 (54%)
T2	87 (34%)
T3	28 (11%)
LN metastasis	
Yes	45 (17%)
No	214 (83%)
TNM staging	
0–I	196 (76%)
II	18 (6.9%)
III	44 (17%)
IV	1 (0.4%)
Postoperative outcomes	
Ileus	44 (17%)
Leakage	
All grade^a^/Grade 3 or 4^a^	22 (8.5%)/14 (5.4%)
Reoperation	5 (1.9%)
Bleeding	1 (0.3%)

*Note*: TNM stage is classified by UICC‐7 staging. Values are expressed as median (range). The median tumour distance from the anal verge was 40 mm, and 163 patients (63%) underwent intersphincteric resection.

Abbreviations: AV, anal verge; BMI, body mass index; LN, lymph node.

^a^ CTCAE v5.0.

### QOL score changes over 3 years

**FIGURE 1:**
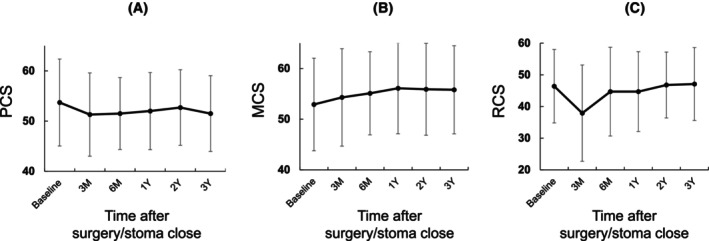
QOL scores changes over 3 years. (A) PCS, physical component summary; (B) MCS, mental component summary; (C) RCS, role/social component summary; QOL, quality of life.

The QOL scores at each time point and their changes over time are presented in Table 2 and Figure 1. The PCS score showed a minor decrease post‐surgery with gradual recovery, the MCS score improved over time, indicating better mental health, and the RCS score initially dropped significantly but showed recovery over the 3‐year follow‐up period.

**TABLE 2:** Quality of life score.

	SF‐36® PCS		SF‐36® MCS		SF‐36® RCS	
	*n*	Mean (SD)	*n*	Mean (SD)	*n*	Mean (SD)
Baseline	259	53.7 (8.65)	259	52.9 (9.14)	259	46.4 (11.6)
3 months	209	51.3 (8.29)	209	54.3 (9.62)	209	37.9 (15.2)
6 months	197	51.4 (7.16)	197	55.1 (8.21)	197	42.6 (14.0)
1 year	182	52.0 (7.68)	182	56.1 (8.97)	182	44.7 (12.6)
2 years	151	52.7 (7.53)	151	55.9 (9.07)	151	46.7 (10.4)
3 years	137	51.9 (7.54)	137	55.8 (8.69)	137	47.1 (11.5)

Abbreviations: MCS, mental component summary; PCS, physical component summary; RCS, role/social component summary.

### Subgroup analysis

**FIGURE 2:**
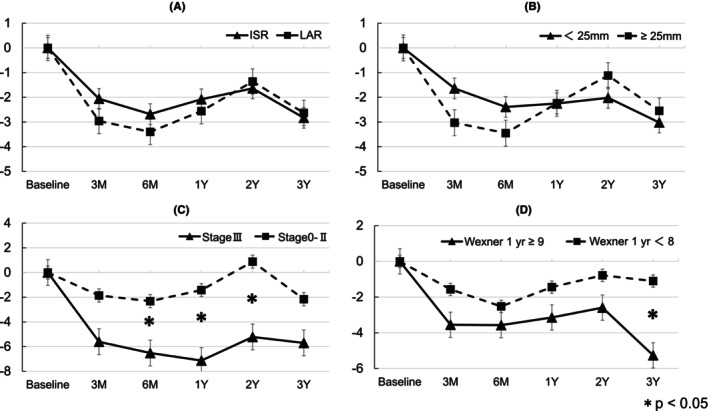
Subgroup analysis of PCS. (A) Surgical method (ISR vs. LAR), (B) tumour distance from AV (<25 mm vs. ≥25 mm), (C) pathological stage (Stage III vs. Stages 0–II), (D) Wexner score at 1 year (≥9 vs. <8). The difference between the two groups was statistically significant (*p < 0.05). AV, anal verge; ISR, intersphincteric resection; LAR, low anterior resection; PCS, physical component summary.

**FIGURE 3:**
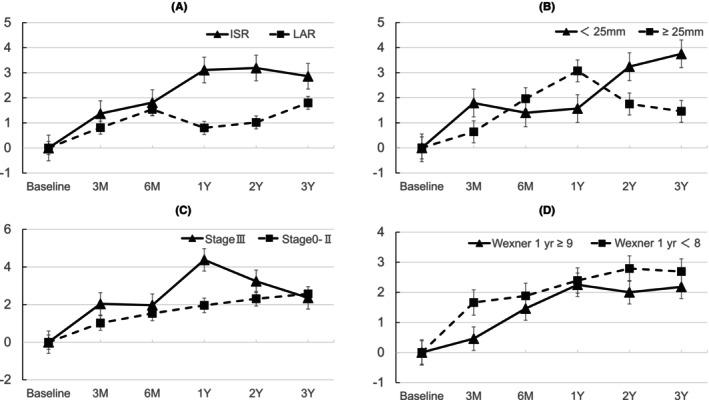
Subgroup analysis of MCS. (A) Surgical method (ISR vs. LAR), (B) tumour distance from AV (<25 mm vs. ≥25 mm), (C) pathological stage (Stage III vs. Stages 0–II), (D) Wexner score at 1 year (≥9 vs. <8). AV, anal verge; ISR, intersphincteric resection; LAR, low anterior resection; MCS, mental component summary.

**FIGURE 4:**
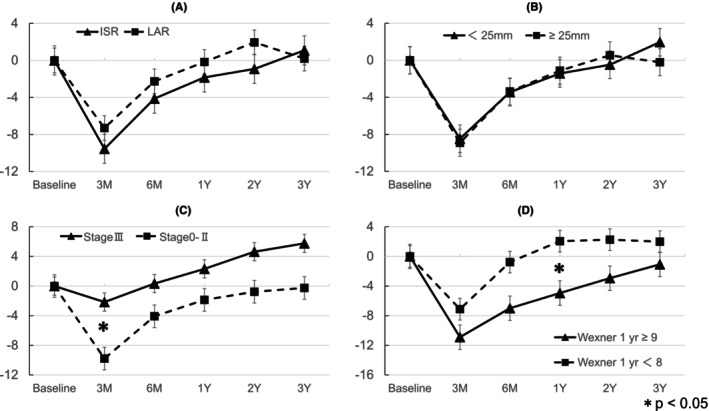
Subgroup analysis of RCS. (A) Surgical method (ISR vs. LAR), (B) tumour distance from AV (<25 mm vs. ≥25 mm), (C) pathological stage (Stage III vs. Stages 0–II), (D) Wexner score at 1 year (≥9 vs. <8). The difference between the two groups was statistically significant (*p < 0.05). AV, anal verge; ISR, intersphincteric resection; LAR, low anterior resection.

The PCS, MCS, and RCS scores over time across the different patient subgroups are shown in Figures 2, 3, 4. Patients with stage III cancer showed the most pronounced decline in the PCS. Patients with stage III cancer had significantly lower PCS scores than did patients with stage 0–II cancers at 6 months, 1 year, and 2 years. The MCS scores improved over time across all subgroups. Regarding the MCS score, no significant differences were found at any time point. The RCS scores were lower at 3 months than at baseline but gradually recovered over time. Significantly lower RCS scores were observed in patients with stage III cancer versus those with stage 0–II cancer at 3 months and in patients with Wexner score ≥9 at 1 year versus those with Wexner score <8 at 1 year.

### Multivariate analysis

Multivariate analysis identified stage III cancer as a significant risk factor for PCS decline (*p* = 0.0084) and a Wexner score >9 at 1 year as a risk factor for RCS decline (*p* = 0.0116) (Table 3).

**TABLE 3:** Multivariate analysis using a mixed effects model. (a) Physical component summary, (b) Mental component summary, (c) Role/social component summary.

Effect	Estimate	Standard error	*t* Value	*p*	Lower 95% CI	Upper 95% CI
(a)						
ISR	0.8257	0.8193	1.01	0.3138	0.7824	2.4338
Stage III	2.6726	1.0114	2.64	0.0084*	0.6874	4.6577
Wexner 1 year ≥9	1.3632	0.8261	1.65	0.0993	0.2583	2.9847
(b)						
ISR	0.9191	0.8606	1.07	0.2859	0.7702	2.6083
Stage III	0.3349	1.0625	0.32	0.7527	1.7505	2.4203
Wexner 1 year ≥9	0.7176	0.8677	0.83	0.4084	0.9854	2.4207
(c)						
ISR	0.1665	1.439	−0.12	0.9079	2.6579	2.9909
Stage III	2.8902	1.7758	1.63	0.104	0.5953	6.3757
Wexner 1 year ≥9	3.673	1.4528	2.53	0.0116*	0.8216	6.5247

Abbreviations: CI, confidence interval; ISR, intersphincteric resection.

* Significant difference: *p* < 0.05.

## DISCUSSION AND CONCLUSIONS

This study evaluated the QOL of patients who underwent laparoscopic surgery for very low rectal cancer located near the anus with a 3‐year follow‐up using the SF‐36 questionnaire. These findings revealed important trends in QOL recovery over time.

The PCS score initially declined postoperatively and gradually recovered over time. However, it is important to note that, even at 3 years postoperatively, the PCS score remained lower than the baseline level. This suggests that, although physical function improved, patients may have continued to experience some degree of long‐term impairment. Additionally, patients with stage III cancer experienced a more significant and prolonged reduction in the PCS score, compared with those with stages 0–II cancer. Multivariate analysis identified stage III cancer as a significant risk factor for PCS decline (*p* = 0.0084). This highlights the substantial effect of advanced cancer and its treatments on physical health. The greater decline in the PCS score in patients with stage III cancer may be attributed to the adjuvant chemotherapy. Of the 259 patients analysed in this study, 58 were pathologic stage III. Among these, 43 patients received adjuvant chemotherapy. Common regimens, such as capecitabine and oxaliplatin, are known to have adverse effects. Capecitabine frequently causes hand‐foot syndrome, whereas oxaliplatin often leads to peripheral neuropathy, a persistent condition that can significantly affect long‐term QOL. Similar findings were reported by Malik et al. [18] in their study of stages I–III colon cancer survivors. Their nationwide retrospective cohort study using the GIQLI and SF‐36 questionnaires revealed that patients receiving adjuvant chemotherapy had significantly impaired QOL, with oxaliplatin‐induced neurotoxicity being the primary factor. In our study, the observation that patients with stage III cancer exhibited a significant decline in the PCS score beyond 6 months post‐surgery, compared with patients with stage 0–II cancer, suggests that adjuvant chemotherapy may adversely affect physical health recovery. These results, combined with ours, emphasize the need to optimize chemotherapy regimens to balance treatment effectiveness with QOL. Strategies for achieving this balance include reducing the number of chemotherapy cycles and adopting protocols for managing neurotoxicity. The IDEA trial [19] supports these approaches, showing that a shorter, 3‐month course of oxaliplatin‐based therapy can significantly reduce the risk of peripheral neuropathy, compared with a 6‐month course regimen while maintaining comparable disease‐free survival for low‐risk patients. These results suggest a more personalized approach to treatment, tailoring the chemotherapy duration to each patient's risk profile, to improve long‐term QOL while ensuring effective cancer control. Furthermore, subgroup analyses revealed no significant differences in the PCS score based on the surgical method (ISR vs. LAR) or distance of the tumour from the AV (<25 mm vs. ≥25 mm). Thus, future studies should focus on minimizing the side effects of chemotherapy early in the treatment process. Multidisciplinary teams, including oncologists, physical therapists, and psychologists, are crucial for providing comprehensive support during recovery. Looking ahead, the systematic use of patient‐reported outcomes in follow‐up care may support personalized treatment strategies, leading to improved long‐term outcomes for cancer survivors.

In contrast to the PCS score, the MCS score consistently increased from baseline. This trend indicated that patients experienced greater mental stability after undergoing cancer surgery than at the preoperative baseline. This result is consistent with previous findings that psychological well‐being often stabilizes following surgery [14]. Although the multivariate analysis did not identify significant risk factors for decline in the MCS score, recovery outcomes may differ among individuals and may be influenced by factors such as family support, financial security and personal coping strategies.

The RCS score declined sharply at 3 months postoperatively but exhibited progressive recovery throughout the follow‐up period. A plausible explanation for the sharp decline in the RCS score is the difference in psychological adaptation processes during the early recovery period. Although physical discomfort and fatigue, which are reflected in the PCS, are expected after major surgery, patients may anticipate these issues and be mentally prepared for physical recovery. Similarly, the MCS may remain stable or even improve over time owing to the relief of cancer‐related anxiety following successful surgical intervention. However, the RCS is heavily dependent on how well patients can reintegrate into their daily social and professional roles, and this aspect of recovery may not align with their expectations. Many patients assume that surgery will resolve cancer‐related concerns and allow them to resume their normal activities within a short timeframe. Bowel dysfunction after rectal surgery significantly restricts patients' ability to work, travel or participate in social events, which may lead to frustration, social withdrawal and loss of confidence in their postoperative recovery. This psychological mismatch, in which patients feel physically capable but socially restricted, may be a key factor contributing to the pronounced decline in the RCS. Multivariate analysis identified bowel dysfunction (Wexner score ≥9 at 1 year) as a significant risk factor for lower RCS score (*p* = 0.0116). These results highlight the prolonged impact of postoperative bowel dysfunction on patients' social and role functioning. Unlike physical recovery, which generally improves over time, bowel dysfunction can persist and be unpredictable, leading to long‐term restrictions in daily life. Previous studies have reported that stoma patients exhibit better social functioning than non‐stoma patients do, experiencing less anxiety and higher self‐esteem [20]. This suggests that, despite physical limitations, stable bowel function may facilitate social reintegration. For patients with severe social impairment, stoma creation could be a potential solution to improve their social QOL.

It is important to note that, although the protocol required tumours to be within 50 mm of the anal verge, intra‐operative re‐measurement under anaesthesia showed that two patients actually had tumours at 55 mm. This happened because pre‐operative measurements, taken without anaesthesia, can sometimes underestimate the actual distance from the anal verge. To ensure accuracy, we confirmed all distances intra‐operatively. We acknowledge this small discrepancy here to avoid any misunderstanding of our tumour location data.

Despite the valuable insights gained from this study, some limitations must be acknowledged. First, this study only included patients who met strict inclusion criteria (T1–T2, N0 and M0 tumours located within 5 cm of the AV); therefore, the findings may not be generalizable to patients with more advanced disease or different tumour locations. Additionally, patients with severe comorbidities were excluded, leading to a possible overestimation of the post‐operative recovery outcomes. Second, many patients with stage III cancer underwent adjuvant chemotherapy, and the specific impacts of different chemotherapy regimens on QOL were not analysed separately. Future studies should differentiate between the effects of surgery, chemotherapy and other adjuvant treatments to better guide treatment decisions and optimize supportive care strategies. Third, tumour recurrence is a critical factor that can profoundly affect the QOL; however, this study did not incorporate recurrence status into the analysis. As noted in the primary study, the 3‐year cumulative local recurrence rate was 6.3% (95% confidence interval [CI]: 3.9–9.5), and the 3‐year recurrence‐free survival and overall survival rates were 83.6% (95% CI: 78.6–87.5) and 94.1% (95% CI: 90.3–96.4), respectively. However, in this sub‐analysis, some patients' questionnaire response timings were unclear, making it difficult to assess the direct impact of tumour recurrence on the QOL outcomes. Fourth, there was a substantial proportion of patients lost to follow‐up at 3 years. We attempted to categorize reasons for dropout, but due to the multi‐institutional design, not all reasons could be collected systematically. To address this, we compared patients who completed all follow‐up assessments with those who did not and found no significant differences in baseline or key clinical factors. The details of this comparison are provided in Table S1. While this comparison supports the validity of our conclusions, we acknowledge that the high dropout rate remains a limitation. Finally, this study was conducted as a prospective, non‐randomized, single‐arm phase II trial. The lack of a control group limited our ability to compare the long‐term QOL outcomes of laparoscopic surgery with those of other surgical approaches, such as open or robot‐assisted surgery.

In conclusion, this study indicates that the QOL after laparoscopic surgery for low‐lying rectal cancer is generally acceptable. The PCS score initially declined post‐surgery and gradually recovered over time, the MCS score demonstrated a steady improvement, and the RCS score exhibited an initial decline at 3 months but showed recovery over 3 years. A key strength of this study is its prospective design with long‐term follow‐up, allowing for a comprehensive evaluation of not only the physical but also the mental and role/social aspects of QOL. Patients with stage III cancer experience prolonged physical challenges, whereas those with bowel dysfunction struggle with the social aspects of QOL. Clinicians should recognize that patients with stage III disease may require additional interventions to support physical recovery after surgery.

## AUTHOR CONTRIBUTIONS

**Hiroki Hamamoto:** Writing – original draft; conceptualization; methodology. **Keitaro Tanaka:** Writing – review and editing; conceptualization; methodology; supervision. **Yuichiro Tsukada:** Writing – review and editing; conceptualization; methodology; data curation; project administration; supervision. **Jun Watanabe:** Writing – review and editing. **Yosuke Fukunaga:** Writing – review and editing. **Yasumitsu Hirano:** Writing – review and editing. **Kazuhiro Sakamoto:** Writing – review and editing. **Masanori Yoshimitsu:** Writing – review and editing. **Hisanaga Horie:** Writing – review and editing. **Nobuhisa Matsuhashi:** Writing – review and editing. **Yoshiaki Kuriu:** Writing – review and editing. **Shuntaro Nagai:** Writing – review and editing. **Madoka Hamada:** Writing – review and editing. **Shinichi Yoshioka:** Writing – review and editing. **Shinobu Ohnuma:** Writing – review and editing. **Tamuro Hayama:** Writing – review and editing. **Koki Otsuka:** Writing – review and editing. **Yusuke Inoue:** Writing – review and editing. **Kazuki Ueda:** Writing – review and editing. **Yuji Toiyama:** Writing – review and editing. **Satoshi Maruyama:** Writing – review and editing. **Shigeki Yamaguchi:** Writing – review and editing. **Motoko Suzuki:** Data curation; writing – review and editing; methodology; formal analysis. **Toshihiro Misumi:** Data curation; writing – review and editing; methodology; formal analysis. **Takeshi Naitoh:** Writing – review and editing. **Masahiko Watanabe:** Writing – review and editing. **Masaaki Ito:** Writing – review and editing; data curation; project administration; supervision.

## FUNDING INFORMATION

The authors have no related conflicts of interest to declare.

## CONFLICT OF INTEREST STATEMENT

The authors have no conflicts of interest directly relevant to the content of this article.

## ETHICS STATEMENT

This prospective study was approved by the Ethics Committee of the Japanese Society for Cancer of the Colon and Rectum and was approved by the institutional review board of each participating hospital or university.

## CONSENT

Written informed consent was obtained from all participants.

## Supporting information


Figure S1.

Table S1.

## Data Availability

The data sets generated and/or analysed during the current study are not publicly available. However, they are available from the corresponding author upon reasonable request.
