# Supplementary material for: Quality of life after laparoscopic surgery for very low rectal cancer: A sub‐analysis of the ultimate trial
Source: Colorectal Dis. 2025 Oct 6;27(10):e70255. doi: 10.1111/codi.70255 (PMC12501488; doi:10.1111/codi.70255)
Supplement: Supplementary file 1 — Figure S1. [file CODI-27-0-s001.pptx]

## Slide 1
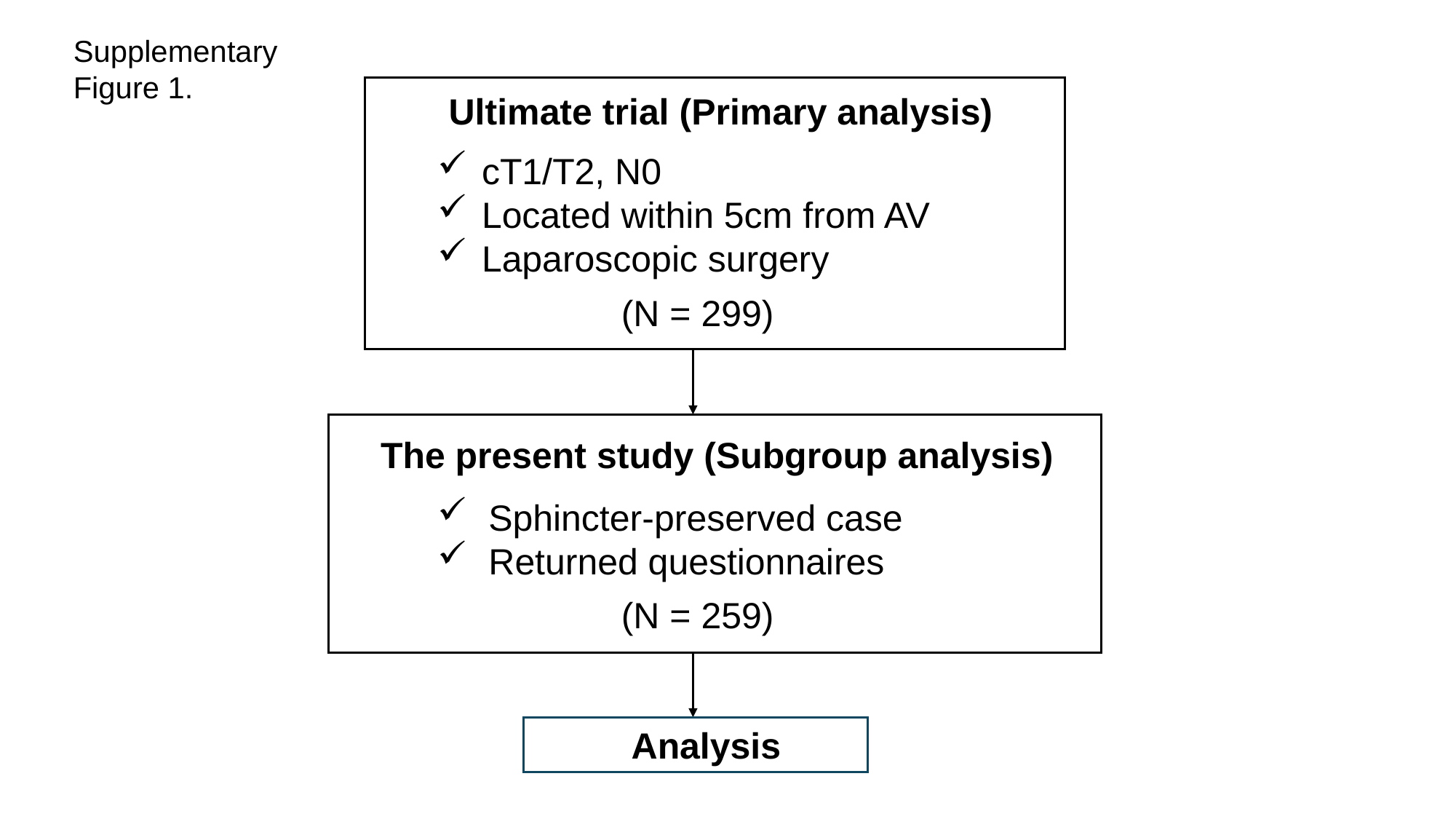

Supplementary Figure 1.
Ultimate trial (Primary analysis)
 cT1/T2, N0
 Located within 5cm from AV
 Laparoscopic surgery
(N = 299)
The present study (Subgroup analysis)
 Sphincter-preserved case
 Returned questionnaires
(N = 259)
Analysis
