# Supplementary material for: Quality of life after laparoscopic surgery for very low rectal cancer: A sub‐analysis of the ultimate trial
Source: Colorectal Dis. 2025 Oct 6;27(10):e70255. doi: 10.1111/codi.70255 (PMC12501488; doi:10.1111/codi.70255)
Supplement: Supplementary file 2 — Table S1. [file CODI-27-0-s002.docx]

Supplementary Table 1.

Comparison of baseline characteristics and key factors between patients with complete follow-up and patients with incomplete follow-up.

|  | Patients with complete  follow-up (n = 137) | Patients with incomplete  follow-up (n = 122) | p |
| --- | --- | --- | --- |
| Sex  (Male / Female) | 79 / 58 | 83 / 39 | 0.08 |
| Age | 64 (30-84) | 64 (35-87) | 0.44 |
| Tumor from AV (mm) | 40 (10-55) | 40 (10-55) | 0.84 |
| Clinical T stage  (T1 / T2)) | 83 / 54 | 69 / 53 | 0.51 |
| ISR operation | 88 (64%) | 74 (61%) | 0.55 |
| Diverting stoma | 127 (93%) | 111 (91%) |  |
| Final Stage  (0-I / II / III / IV) | 101 / 9 / 27 / 0 | 95 / 9 / 17 / 1 | 0.07 |
| Leakage | 9 (6.6%) | 13 (11%) | 0.24 |

ISR: Intersphincteric resection. TNM stage is classified by UICC-7 staging. Values are expressed as median (range).
